# Pertrochanteric hip fracture is associated with mobility decline and poorer physical performance 4 to 6 months post-hip fracture

**DOI:** 10.1186/s12877-023-04415-x

**Published:** 2023-11-08

**Authors:** Minna A. Kujala, Markus T. Hongisto, Tiina Luukkaala, Sari Stenholm, Maria S. Nuotio

**Affiliations:** 1 Department of Geriatric Medicine, Seinäjoki Central Hospital, Wellbeing Services County of South Ostrobothnia, Seinäjoki, Finland; 2 Department of Geriatric Medicine, University of Turku, Turku, Finland; 3 Division of Orthopaedics and Traumatology, Seinäjoki Central Hospital, Wellbeing Services County of South Ostrobothnia, Seinäjoki, Finland; 4 Faculty of Medicine and Health Technology, Tampere University, Tampere, Finland; 5 Research and Innovation Centre, Tampere University Hospital, Tampere, Finland; 6 Health Sciences, Faculty of Social Sciences, Tampere University, Tampere, Finland; 7 Department of Public Health, University of Turku and Turku University Hospital, Turku, Finland; 8 Centre for Population Health Research, University of Turku and Turku University Hospital, Turku, Finland; 9 Research Services, Turku University Hospital and University of Turku, Turku, Finland; 10 Department of Geriatric Medicine, University of Turku and Turku University Hospital, Turku, Finland

**Keywords:** Geriatric assessment, Hip fracture, Femoral neck fracture, Pertrochanteric fracture, Subtrochanteric fracture, Orthogeriatric care, Physical performance, Mobility, Disability, Muscle strength

## Abstract

**Background:**

To study the effect of hip fracture type on physical performance, functional ability and change in mobility four to six months after the injury.

**Methods:**

A total of 1331 patients out of consecutive 2052 patients aged ≥ 65 years who underwent hip fracture surgery were included in the study. Patient information was collected on admission, during hospitalization, by phone interview and at the geriatric outpatient clinic 4 to 6 months after the fracture. Of the 1331 eligible patients, Grip strength, Timed Up and Go -test (TUG), Elderly Mobility Scale (EMS), mobility change compared to pre-fracture mobility level, Basic Activities of Daily Living (BADL) and Instrumental Activities of Daily Living (IADL) were used to determine physical performance and functional ability. Logistic regression was used for the analyses which was adjusted for gender, age, American Society of Anesthesiologists score, diagnosis of cognitive disorder, pre-fracture living arrangements, mobility and need of mobility aid.

**Results:**

Patients with pertrochanteric hip fracture had an EMS lower than 14 (Odds Ratio (OR) 1.38, 95% confidence intervals (CI) 1.00–1.90), TUG time ≥ 20 s (OR 1.69, 95% CI 1.22–2.33) and they had declined in mobility (OR 1.58, 95% CI 1.20–2.09) compared to femoral neck fracture patients 4 to 6 months post-hip fracture in multivariable-adjusted logistic regression analyses. Grip strength and functional ability (IADL, BADL) 4 to 6 months after hip fracture did not differ between fracture types. There were no statistically significant differences in physical performance in patients with a subtrochanteric fracture compared to patients with a femoral neck fracture.

**Conclusions:**

Pertrochanteric hip fracture independently associated with poorer physical performance 4 to 6 months post hip fracture compared to other hip fracture types. Pertrochanteric hip fracture patients should be given special attention in terms of regaining their previous level of mobility.

**Supplementary Information:**

The online version contains supplementary material available at 10.1186/s12877-023-04415-x.

## Background

Hip fracture is a common and severe consequence of a fall for older people. Hip fractures often lead to a decline in mobility, a lower quality of life and increased need for assistance in activities of daily living [1, 2]. The excess mortality of hip fracture patients is highest during the first year after the hip fracture and remains elevated several years thereafter [3, 4]. Age-adjusted incidence of hip fractures is declining in Finland, but the number of hip fractures will increase due to a sharp growth of the aging population in the upcoming decades [5].

Hip fractures are classified according to anatomic location of the fracture in the upper femur and in relation to the hip capsule. The most common fracture type is femoral neck fracture (intracapsular), which accounts for 60% of all fractures. Pertrochanteric fractures account for 30% of all fractures and subtrochanteric fractures 10% [2]. Hip fracture type and displacement of fracture defines the method of surgical treatment. Intracapsular and extracapsular hip fractures may also have different risk factors and sequelae. For example, the proportion of pertrochanteric fractures increases with age in women, but in men the proportion of pertrochanteric fractures falls or remains the same [6, 7]. Decline in mobility and function is common after a hip fracture. In a review by Dryer et al., 34–59% of patients regained basic ADL function by three months, and 42–71% by six months [1]. However, relatively few studies have analysed different hip fracture types separately. To the best of our knowledge, studies on potential differences in short-term and long-term physical function and mobility in particular are limited. Some studies report comparable functional outcomes in patients with pertrochanteric fractures and femoral neck fracture one year after the hip fracture[8, 9]. Pertrochanteric and subtrochanteric fractures are associated with a higher need for postoperative blood transfusions, a higher mean operation time and a longer length of hospital stay [10, 11]. Femoral neck fractures are associated with higher readmissions and reoperation rates [11]. Fewer problems in everyday life in aspects of mobility, self-care, usual activities, pain and mental health, a higher quality of life, and lower average pain have been reported in patients with femoral neck fractures compared to those with trochanteric or subtrochanteric fractures in a post fracture assessment [12]. The varying outcomes of hip fracture patients depend on patient selection and treatment methods. However, there may be differences in baseline characteristics across hip fracture types that can influence the outcomes measured. It is worth noting that study reports including subtrochanteric fractures analyzed separately, are limited.

In several hip fracture studies, pertrochanteric fractures and subtrochanteric fractures are categorized to represent extracapsular fractures. Compared to patients with extracapsular hip fractures, intracapsular hip fracture patients are more likely to be men, have a higher level of education, live with a caregiver, exhibit higher functional levels on admission and upon discharge, have higher cognitive functions and require shorter rehabilitation time. In addition, intracapsular hip fracture patients have a longer latency time from fracture to surgery, tend to be younger, healthier on hospital admission and have a shorter hospital stay than extracapsular fracture patients [13, 14]. However, in a study of 170 female hip fracture patients, there were no significant differences between the two groups with respect to median age (80 and 78 years, respectively), type and number of comorbidities and pre-fracture residence at the time of the injury [9].

Subtrochanteric fractures are markedly less common than femoral neck and pertrochanteric fractures. Powerful muscle groups attach to proximal femur and fracture displacement may cause challenges in the hip fracture operation. Two distinct small subgroups of patients of subtrochanteric fractures have been observed in previous studies. Subtrochanteric fractures are more common in young men who sustain high-energy trauma and in osteoporotic women who have had bisphosphonate treatment for more than 5 years [15, 16].

All hip fracture patients should be rehabilitated targeting their previous level of mobility and functional capabilities. However, there is a need to identify patients who are at increased risk of declining in their physical function and functional ability so that the intensity of limited rehabilitation resources can be targeted to patients who are in most need and likely to benefit from them. Hip fracture type may be one of the underlying factors predicting recovery and functional development after the hip fracture.

Therefore, our research aimed to study the association of different hip fracture types on physical performance, change in mobility and functional ability 4 to 6 months after the hip fracture.

## Methods

### Study population

This present study comprised a prospectively documented cohort including 2 052 consecutive hip fracture patients aged ≥ 65 who sustained their first hip fracture between September 2007 and January 2019 in the Hospital District of Southern Ostrobothnia, Finland. Basically, all patients who suffer from a hip fracture or surgical complication after treatment of a hip fracture inside the referral area are admitted and operated at Seinäjoki Central Hospital [17]. Patients who had a pathological or periprosthetic hip fracture were excluded from the study.

**Fig. 1:**
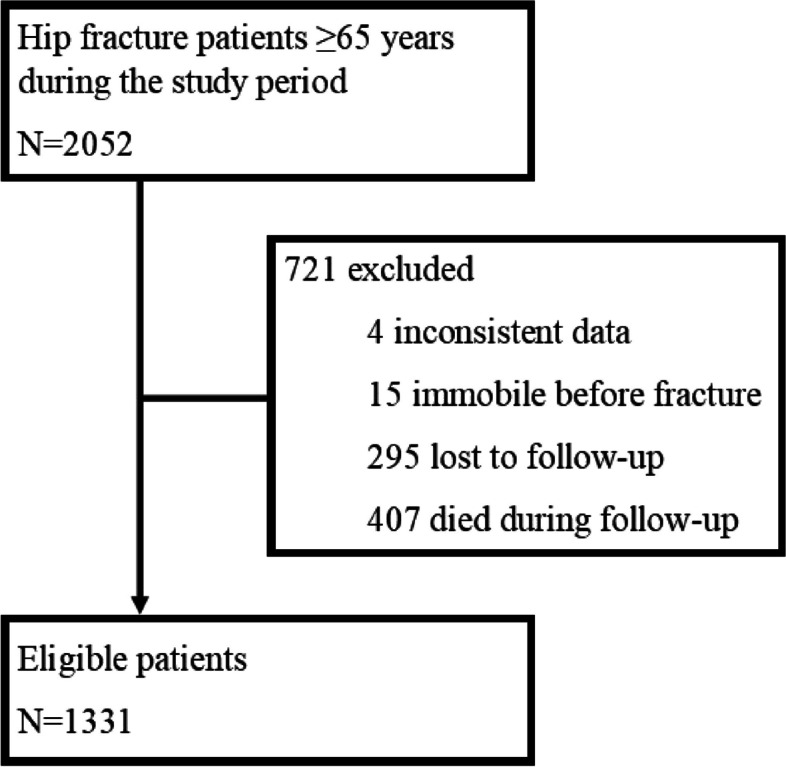
Flow chart of study population

The target time for the follow-up visit was 4 months after the fracture, but due to the waiting list situation and patient related factors, the appointment time was realized 4 to 6 months after the fracture. Before the follow-up visit, 407 patients (20%) had died. Of those patients who were still alive, 1331 patients (81%) with the necessary documentation of variables attended the follow-up visit and were included in the study (Fig. 1).

### Data collection

In 2007, a program for orthogeriatric care was launched along with a prospective data collection [17]. Patient characteristics or clinical tests were collected on admission, during hospitalization, by phone interview and 4 to 6 months after the fracture at the geriatric outpatient clinic by a multidisciplinary team. If a patient was incapable of providing information due to their health condition or cognitive problems, family members, close friends, or nurses from a health care facility were used to obtain the data. During the study period, predefined inquiries modified from British hip fracture register were used to obtain as accurate data as possible [18]. Data has been collected over several years. More measures of physical performance have been added to the outpatient visit during the data collection and therefore reports with different performance tests were available for a varying number of patients. Information on changes in mobility was collected by phone interviews 4 months after the fracture. Follow-up visits at the geriatric outpatient clinic with a comprehensive geriatric assessment (CGA) was carried out 4 to 6 months after hip fracture by a multidisciplinary team [19]. A physiotherapist´s examination including the physical performance tests preceded the geriatric assessment. Both patient and his or her next of kin or caregiver were invited.

On admission patients or their representatives were asked to give their informed consent for the data collection. The study design was approved by the Ethics Committee of the Hospital District of South Ostrobothnia. The Strobe reporting guidelines were followed.

### Fracture types, surgical methods, and baseline characteristics

Fracture diagnoses of the upper femur set by the orthopaedic and trauma surgeon were derived from the electronic patient files and were categorized as femoral neck fracture, pertrochanteric fracture and subtrochanteric fracture.

Among all patients with a femoral neck fracture, 83% were treated with hemiarthroplasty (HA), 13% with total hip arthroplasty (THA) and 4% with internal fixation. Physiologically exceptionally active and mobile femoral neck fracture patients were treated with total hip arthroplasty. The surgeon on duty decided whether to use a lateral (modified Hardinge) or posterior approach for the repair to the posterior capsule and external rotators in both HA and THA. The implant used in the HA was uncemented or cemented modular monopolar prosthesis. Internal fixation was used only in Garden I and II fractures with good bone quality. However, when a patient had severe osteoporosis or co-morbidities adversely affecting bone healing, HA was used even in Garden II fractures. Stable pertrochanteric fractures were treated with a short intramedullary nail with a sliding screw, whereas unstable pertrochanteric and subtrochanteric fractures were treated with a long intramedullary nail with a sliding screw.

Nutritional status was measured using the Mini-Nutritional Assessment-Short Form (MNA-SF), which is a short screening tool for nutritional status with documented clinical relevance and validation in older populations [20]. The MNA-SF was categorized into three groups: normal [12–14], at risk of malnutrition [8–11] and malnourished (0–7). Living arrangements were categorized as living at home, home with home care, assisted care facility and institution. The number of regularly used medications on admission and American Association of Anaesthesiologists (ASA) scores were used to assess medical co-morbidities [21, 22]. The ASA was categorized into three groups: 1–2,3 and 4–5. Diagnosis of cognitive disorder (yes or no) was registered at the time of fracture. A cognitive disorder known pre-fracture was defined as a clinical diagnosis of cognitive disorder diagnosed by a specialist in geriatric medicine or neurology. The diagnosis was confirmed from the patient records. Mobility level before hip fracture was defined on a basis of survey like questions modified from those originally included in the data collection of the British National Hip Fracture Database. Based on these questions of walking ability, mobility level was graded into 4 groups: 1. Unassisted outdoors, 2. Assisted outdoors, unassisted indoors 3. Assisted indoors and 4. Unable to walk. Mobility level was evaluated by similar questions at baseline and follow-up phone interview at 4 months post-fracture. The need for a mobility aid was also registered. All the baseline variables with their categorizations are listed in Table 1.

**Table 1:** Baseline characteristics of hip fracture patients (*N* = 1331)

	**Femoral neck fracture** ***n*** ** = 839**		**Pertrochanteric fracture** ***n*** ** = 413**		**Subtrochanteric fracture** ***n*** ** = 79**		
	n	(%)	n	(%)	n	(%)	*p*
**Gender**							0.313
Women	613	(73)	317	(77)	61	(77)	
Men	226	(27)	96	(23)	18	(23)	
**Age, years**							< 0.001
65–79	328	(39)	117	(28)	24	(30)	
80–89	420	(50)	231	(56)	37	(47)	
≥ 90	91	(11)	65	(16)	18	(23)	
**MNA-SF**							0.097
Normal nutrition 12–14	391	(47)	191	(46)	30	(38)	
At risk of malnutrition 8–11	211	(25)	118	(29)	30	(38)	
Malnourished 0–7	22	(3)	14	(3)	4	(5)	
Unknown	215	(25)	90	(22)	15	(19)	
**Living arrangements**							0.006
Home	452	(54)	177	(43)	41	(52)	
Home with home care	220	(26)	147	(36)	18	(23)	
Assisted care facility	80	(9)	38	(9)	6	(8)	
Institution	83	(10)	50	(12)	13	(17)	
**ASA**							0.028
Grade 1–2	166	(20)	52	(13)	11	(14)	
Grade 3	514	(61)	291	(70)	55	(70)	
Grade 4–5	148	(18)	63	(15)	12	(15)	
Unknown	11	(1)	7	(2)	1	(1)	
**Number of medication**							0.654
< 4	173	(21)	71	(17)	15	(19)	
4–10	536	(64)	271	(66)	50	(63)	
> 10	130	(15)	71	(17)	14	(18)	
**Diagnosis of** **cognitive disorder**							0.255
No	651	(78)	304	(74)	56	(71)	
Yes	186	(22)	109	(26)	23	(29)	
**Mobility level before hip fracture**							0.181
Unassisted outdoors	556	(66)	258	(62)	50	(63)	
Assisted outdoors,unassisted indoors	246	(29)	139	(34)	25	(32)	
Assisted indoors	30	(4)	11	(3)	1	(1)	
Unable to walk	7	(1)	5	(1)	3	(4)	
**Need of mobility aid**							< 0.001
Without mobility aid	413	(49)	147	(36)	37	(47)	
Needs mobility aid or unable to walk	426	(51)	266	(64)	42	(53)	

Differences between fracture types were tested using Pearson Chi-Square test or Fisher-Freeman-Halton Exact test. Unknown results of the variables less than 10 was excluded from the table. *MNA-sf* Mini Nutritional Assessment short form, *ASA* The American Society of Anaesthesiologists’ classification of physical health

### Physical performance and functional ability

Primary outcome variables were grip strength, Timed Up and Go -test (TUG), Elderly Mobility Scale (EMS), Basic Activities of Daily Living (BADL), Instrumental Activities of Daily Living (IADL) and mobility change. Physical performance was measured with grip strength, TUG and EMS whereas IADL and BADL describe functional ability.

A physiotherapist´s examination preceding the geriatric outpatient clinic visit included a hand grip test, a TUG and an EMS. The hand grip strength is an easy and inexpensive method to assess muscle strength. Grip strength correlates moderately with strength in other body parts and was measured using a Jamar handheld dynamometer. Impaired grip strength for women was considered to be < 16 kg and for men < 27 kg. The definition for impaired grip strength and impaired TUG were chosen according to the 2019 update on the European Working group on Sarcopenia in Older People (EWGSOP2) [23, 24].

The TUG test is a modified, timed version of the “Get-Up and Go” test [25]. The test requires patients to stand up from a chair, walk 3-m distance, turn around, return, and sit down again. Timed up and go test measures physical performance and predicts falls and has been used to identify frail older individuals [26]. We categorized normal cut off point as TUG ≥ 20 s [23].

The EMS is a standardized validated scale for assessing the mobility of frail older people[27] EMS is also easy to perform on patients with cognitive impairment and mainly measures functional mobility [28]. Patients who score 14 or more are able to perform the mobility manoeuvres alone and safely and this group of patients is independent in basic ADL [27] The movements of the EMS test include lying to sitting, sitting to lying, sitting to standing, standing, gait, walking speed and functional reach [27]. Based on the practical evaluation, each of the 7 functional tests is awarded a number of points, varying from 0 to 4 and the points are added up. EMS for frail people was categorized as normal (14-20) or abnormal (0–13).

The outcome variable of mobility change was defined as declined mobility from baseline to the 4-months phone interview carried out by the geriatric nurse. The same questions for mobility level, as described in the baseline variables, were used at both time points. Decline in mobility was defined as having more assisted vs. same or less assisted mobility level at follow-up compared to the pre-fracture mobility level.

BADL describes the tasks of everyday life including eating, dressing, getting into or out of a bed or chair, bathing, ability to control movements of the bowel and bladder and using the toilet. Each activity is awarded by one point and points are added up. BADL was categorized as no difficulties (score 6/6), or difficulty in at least one activity [29]. IADL consist of managing communication such as telephone and mail, preparing meals, managing finances, managing transportation, shopping, managing medication, doing laundry, and keeping up home maintenance. IADL was categorized as no difficulties (score 8/8), and difficulties at least in one [30].

### Statistical analyses

The baseline characteristics before fracture and geriatric post fracture assessment after hip fracture were described according to three fracture types as number of patients and percentages of categorical variables. Statistical differences between the groups were tested using Pearson´s chi-square test or Fisher-Freeman-Halton exact test, if appropriate.

Univariable and multivariable adjusted logistic regression analyses was used to examine the association of different hip fracture types for physical performance tests, mobility change and functional ability. The results of patients with pertrochanteric fractures and subtrochanteric fractures were compared with the results of patients with femoral neck fractures. The multivariable analyses were adjusted for gender, age, pre-fracture living arrangements, ASA scores, a known cognitive disorder, mobility and the need for mobility aid. The results of logistic regression analyses are given as odds ratios (OR) with 95% confidence intervals (CIs).

IBM SPSS statistics version 28.0 (SPSS Inc. Chicago, Illinois, USA) was used for statistical analyses. Two-sided p-values under 0.05 were considered statistically significant.

## Results

Median age of the 1331 patients was 83 years (interquartile range 77-87 years), the youngest was 65 and the oldest was 99 years. The majority of participants were women (74%). Of the patients, 63% had a femoral neck fracture, 31% had a pertrochanteric fracture and 6% had a subtrochanteric fracture. Median time for follow-up visit to the geriatric outpatient clinic for femoral neck fracture and pertrochanteric fracture patients was 5 months (interquartile range 4–7 months) and for subtrochanteric fracture patients 6 months (interquartile range 5–7 months).

Table 1 shows the baseline characteristics of hip fracture patients according to the hip fracture type. There were differences between hip fracture types in age, living arrangements, ASA grade and the need for a mobility aid. The proportion of the youngest age group 65–79-year-old patients was highest in the femoral neck fracture patients. The pertrochanteric fracture patients had the lowest proportion of patients living at home and the need of home care and a mobility aid was more common in the pertrochanteric fracture patients than in the femoral neck fracture and the subtrochanteric fracture patients. There was no statistically significant difference in the distribution of gender, results on the MNA-SF, diagnosis of cognitive disorder or number of medications in patients with different fracture types.

Declining in physical performance, mobility and functional ability occurred most frequently in pertrochanteric fracture patients. (Table 2) The results of logistic regression analyses of the association of different hip fracture types and physical performance, mobility change, and functional ability are shown in Table 3. In unadjusted models, patients with a pertrochanteric fractures had low grip strength (OR 1.39, 95%CI 1.01–1.91), TUG time ≥ 20 s (OR 1.98, 95%CI 1.5–2.62), EMS < 14 (OR 1.61, 95% CI 1.24–2.09), a decline in mobility (OR 1.86, 95% CI 1.44–2.39), BADL 0–5 (OR 1.57, 95%CI 1.22–2.03) and IADL 0–7 (OR 1.89, 95% CI 1.32–2.70) compared to patients with femoral neck fractures. The patients with subtrochanteric fractures showed no differences in terms of the aforementioned variables compared to patients with a femoral neck fracture.

**Table 2:** Physical performance, mobility and functional ability at 4–6 months after hip fracture by fracture type (*N* = 1331)

	**Neck of femur fracture ** ***n*** ** = 839**		**Pertrochanteric fracture** ***n*** ** = 413**		**Subtrochanteric fracture** ***n*** ** = 79**		
	**n**	**(%)**	**n**	**(%)**	**n**	(%)	*p*
**Grip strength**							0.087
Men ≥ 27kg, women ≥ 16kg	184	(22)	76	(19)	13	(17)	
Men < 27kg, Women < 16kg	369	(44)	212	(51)	43	(54)	
Unknown	286	(34)	125	(30)	23	(29)	
**Timed Up and Go**							< 0.001
0–19	320	(38)	98	(24)	27	(34)	
20 to highest	395	(47)	240	(58)	40	(51)	
Unknown	124	(15)	75	(18)	12	(15)	
**Elderly Mobility Scale**							0.006
14–20	580	(69)	241	(58)	51	(65)	
0–13	211	(25)	141	(34)	23	(29)	
Unknown	48	(6)	31	(8)	5	(6)	
**Change of mobility**							< 0.001
Same or better	512	(61)	190	(46)	41	(52)	
Declined	260	(31)	179	(43)	28	(35)	
Unknown	67	(8)	44	(11)	10	(13)	
**Basic Activities of Daily Living**							0.004
No difficulties,6	312	(37)	115	(28)	26	(33)	
Difficulties at least in one ≤ 5	509	(61)	295	(71)	52	(66)	
Unknown	18	(2)	3	(1)	1	(1)	
**Instrumental Activities of Daily Living**							0.002
No difficulties, 8	152	(18)	44	(11)	17	(22)	
Difficulties at least in one ≤ 7	668	(80)	365	(88)	61	(77)	
Unknown	19	(2)	4	(1)	1	(1)	

Differences between fracture types were tested using Pearson Chi-Square test

**Table 3:** Univariable and multivariable logistic regression analyses regarding declined physical performance, functional ability and mobility classified by the hip fracture type

**Fracture type**	**Grip strength** *N* = 897 *n* = 624 (70%)		**Timed Up and Go** *N* = 1120 *n* = 675(60%)		**Elderly Mobility Scale** *N* = 1247 *n* = 375 (30%)		**Mobility declined** *N* = 1210 *n* = 467 (39%)		**Basic Activities of Daily Living** *N* = 1309 *n* = 856 (65%)		**Instrumental Activities of Daily Living** *N* = 1307 *n* = 1094 (84%)	
	n	OR (95%CI)	n	OR (95% CI)	n	OR (95% CI)	n	OR (95% CI)	n	OR (95% CI)	n	OR (95% CI)
**Unadjusted**												
Femoral neck	369	1.00	395	1.00	211	1.00	260	1.00	509	1.00	668	1.00
Pertrochanteric	212	1.39 (1.01–1.91)	240	1.98 (1.50–2.62)	141	1.61 (1.24–2.09)	179	1.86 (1.44–2.39)	295	1.57 (1.22–2.03)	365	1.89 (1.32–2.70)
Subtrochanteric	43	1.65 (0.87–3.14)	40	1.20 (0.72–2.00)	23	1.24 (0.74–2.08)	28	1.35 (0.81–2.22)	52	1.23 (0.75–2.00)	61	0.82 (0.46–1.44)
**Multivariable-adjusted**												
Femoral neck	369	1.00	395	1.00	211	1.00	260	1.00	509	1.00	668	1.00
Pertrochanteric	212	1.11 (0.78–1.58)	240	1.69 (1.22–2.33)	141	1.38 (1.00–1.90)	179	1.58 (1.20–2.09)	295	1.21 (0.88–1.66)	365	1.39 (0.91–2.13)
Subtrochanteric	43	1.36 (0.67–2.75)	40	1.13 (0.62–2.06)	23	1.16 (0.60–2.21)	28	1.28 (0.73–2.23)	52	1.02 (0.55–1.90)	61	0.63 (0.31–1.30)

The results for univariable and multivariable adjusted logistic regression were shown by odds ratios (OR) with 95% confidence intervals (CI). Multivariable analyses were adjusted for age, sex, American Association of Anaesthesiologists scores, living arrangements and mobility before fracture, need for mobility aid, diagnosis of cognitive disorder. *N* Number of patients with each test result, *n* Number of patients with low test result. Classification of outcomes: grip strength women < 16kg, men < 27kg vs. more, TUG < 20s vs. ≥ 20, EMS < 14 vs. ≥ 14, mobility level declined from prefracture level (yes vs. no), BADL 0–5 vs. 6, IADL 0–7 vs. 8

In the multivariable analysis adjusted for covariates, a statistically significant association in pertrochanteric fractures remained in TUG (OR 1.69, CI 1.22–2.33), EMS (OR 1.38, 95% CI 1.00–1.90) and a change in mobility (OR 1.58, 95% CI 1.20–2.09). There were no statistically significant differences in functional measures in patients with a subtrochanteric fracture compared to patients with a femoral neck fracture.

We also conducted a drop-out analysis of non-attendees at the geriatric outpatient clinic visit 4 to 6 months after hip fracture. These patients were more likely to be men, were older, had worse nutritional status, were more often living in an institution, general state of health was worse(multimorbid), had multiple medications, had a diagnosis of cognitive disorder, were less often mobile unassisted outdoors and needed mobility aid compared to those who did attend. There was no significant difference in fracture type of attendees and non-attendees. (Supplementary Table 1).

## Discussion

This study demonstrated that patients with a pertrochanteric hip fracture had lower mobility and their mobility had decreased more from their pre-fracture mobility level 4 to 6 months after the fracture than those patients with a femoral neck fracture. There was no statistically significant difference between patients who obtained subtrochanteric fractures and those who acquired femoral neck fractures in terms of losing pre-fracture mobility level, TUG-time, and EMS. Interestingly, no differences in functional ability tests (IADL and BADL) 4 to 6 months after the fracture were observed between hip fracture types.

In this study, a change in mobility 4 to 6 months after the hip fracture differed across hip fracture types. Compared to the baseline situation, the mobility level was the same or better in 61% of femoral neck fracture patients, 52% of subtrochanteric fracture patients and 46% of pertrochanteric fracture patients. This in is line with previous studies where it has been observed that patients with a femoral neck fracture maintained their walking ability and mobility level better after a hip fracture when compared to subtrochanteric fracture and pertrochanteric fracture [31–33]. Most studies group pertrochanteric fractures and subtrochanteric fractures into extracapsular fractures and these fracture types often have similarities. Because of the anatomy, surgical management of a subtrochanteric fracture can be challenging and there may be the risk of a worse prognosis compared to other types of hip fractures [34].

Surprisingly, in our study, the results of the subtrochanteric fracture patients and the femoral neck fracture patients were more similar in the TUG and EMS tests than those with a pertrochanteric fracture. In patients with a subtrochanteric fracture, the better physical outcome compared to patients with a pertrochanteric fracture could be attributed to the location of the fracture, as it does not involve the greater trochanter of the femur. The most important hip abductor muscle is the gluteus medius, which attaches to the greater trochanter of the femur and stabilizes the pelvis in the coronal plane during gait. In pertrochanteric fractures especially, a comminuted fracture in the region of the greater trochanter may lead to increased pain and limping. Previous studies have reported poorer functional outcome in patients with an inter-and/or subtrochanteric fracture compared to cervical fractures and in some studies the fracture type was not predictive of any functional outcomes [8, 9, 35–37].

We did not find association between hip fracture type and low grip strength in multivariable models. In the current study, the cut off points for low strength were < 16 kg for women and < 27 kg for men, which comply with the recommendations of the European working group on Sarcopenia in Older People [23]. A hand grip strength of < 16 kg in women is an independent predictive factor of impaired functional outcome after a hip fracture [38]. It is worth noting that in our study, the majority of patients had low grip strength and there was also a relatively large proportion of missing data. Our study result is in accordance with a previous study concerning women with hip fractures [39]. Grip strength measures upper body strength but does not indicate lower body muscle strength and fractured limb. A hip fracture often leads to reduced muscle strength in the lower limbs. Unfortunately, we did not have information of pre-fracture or post-fracture muscle strength of the lower body, which is important for walking ability.

Fracture incidence increases steeply with increasing TUG time up to 12 s [40]. In our study, very few patients performed well in TUG test. Of the pertrochanteric fracture patients, only 24% of the patients had a TUG of < 20 s and of those with a femoral neck fracture, 38% of the patients had a TUG of < 20 s.

We assessed physical performance using several different measures. EMS has a ceiling effect, but for our study population, EMS was appropriate for assessing the mobility of hip fracture patients.

Functional ability is defined as the ability to carry out activities that require physical actions, including activities of daily living (BADL and IADL). The self-care functions BADL and IADL are likely be affected after hip fracture recovery. In the present study, the fracture type was not a predictor for a poorer BADL and IADL result. This is in accordance with previous studies [41, 42].

The methodological strengths of our study include a large sample with a retrospective analysis of prospectively collected data and the use of well-known and well-validated instruments to measure physical and functional outcomes. In many studies, patients residing in long-term care or patients with dementia are excluded, but in our study, patients in both of these groups were included.

According to the drop-out analysis, the non-attendees at the geriatric outpatient clinic were more likely to be men, were older, had worse nutritional status, were more often living in an institution, general state of health was worse(multimorbid), had multiple medications, had a diagnosis of cognitive disorder, were less often mobile unassisted outdoors and needed mobility aid. If they had been able to attend the follow-up, it is likely that, based on their prefracture status, the results in ADL functions would have been low. However, the effect on physical performance test results and changes in mobility remains unknown.

This study has a number of limitations. First, it is possible that there are differences in hip fracture patient´s baseline characteristics, and the fact that these were not measured may have contributed to our results. Second, our study did not include screening of frailty. Nevertheless, we assessed the patient’s nutritional status according to the MNA-SF, which has been proposed to be used as a potential screening tool for frailty [43]. Third, physical performance or other domains of the CGA, with the exception of eliciting the mobility level, were not assessed at the time of fracture, which means that we could not compare changes in each CGA instrument used at the outpatient clinic. Fourth, our patients went through standard operative procedures and rehabilitation, and we did not assess the impact of any specific type of rehabilitation and functional outcome on different fracture types. Moreover, we did not assess or compare surgical methods on hip fracture repair, which may have had an effect on the outcomes. Hip fracture surgery with good stability and without delay is the key element to a good recovery after the hip fracture. Fifth, we assessed mobility and functional outcome 4 to 6 months after the hip fracture, but a longer follow-up and rehabilitation may also have an effect on these outcomes. This could be addressed in future research. Sixth, the number of subtrochanteric fracture patients was quite small for a multivariable adjusted model which is likely to contribute to the absence of statistically significant results. Finally, as reported in earlier study utilizing same study population and according to the drop-out analysis of the present study, a larger proportion of the patients who did not participate in the follow-up had different patient characteristics for example older age, cognitive disorder, disability and living in more assisted living accommodations [19], which may have had an effect on the outcomes. We do not have complete data on those who decided not to participate in the follow-up but for example in year 2010, 4% of hip fracture patients refused to participate the follow up. Even though we do not have exact numbers for the whole study period, the overall participation rate can be considered high. However, due to missing information, we are unable to include the number of those who refused to follow-up. Patients sustaining a second fracture were not included in the study and unfortunately, we do not have data of patients with second fracture.

## Conclusion

After a pertrochanteric hip fracture, patients are more likely to experience a decline in mobility and a poorer physical performance 4 to 6 months post-fracture than those who suffer from other types of hip fractures. Although mobility performance declines with a pertrochanteric fracture, functional ability does not differ significantly between hip fracture types. Considering these findings, a special focus on supporting the mobility of patients with a pertrochanteric fracture needs to be started from the early postoperative phase; they may also benefit from intensive physically oriented rehabilitation throughout the whole care pathway. In addition, it is important to emphasize that high-quality surgery is a prerequisite for effective rehabilitation.

### Supplementary Information

**Additional file 1: Table 1. **Baseline characteristics of hip fracture patients at the time of fracture among attendees and non-attendees of the geriatric outpatient assessment (*N*=2052).

## Data Availability

The datasets generated and analysed during the current study are not publicly available due to limitations of ethical approval involving the patient data and anonymity but are available from the corresponding author on reasonable request.

## Abbreviations

MNA-sf: Mini Nutritional Assessment short form
ASA score: American Society of Anaesthesiologist score
TUG: Timed Up and Go
EMS: Elderly Mobility Scale
BADL: Basic Activities of Daily Living
IADL: Instrumental Activities of Daily Living
CGA: Comprehensive Geriatric Assessment
HA: Hemiarthroplasty
THA: Total Hemiarthroplasty
