# Supplementary material for: Pertrochanteric hip fracture is associated with mobility decline and poorer physical performance 4 to 6 months post-hip fracture
Source: BMC Geriatr. 2023 Nov 8;23:722. doi: 10.1186/s12877-023-04415-x (PMC10631110; doi:10.1186/s12877-023-04415-x)
Supplement: Supplementary file 1 — Additional file 1: Table 1. Baseline characteristics of hip fracture patients at the time of fracture among attendees and non-attendees of the geriatric outpatient assessment (N=2052). [file 12877_2023_4415_MOESM1_ESM.docx]

**Supplement Table 1. Baseline characteristics of hip fracture patients at the time of fracture among attendees and non-attendees of the geriatric outpatient assessment (N=2052)**

|  | Attendees  n=1331 | | Non-attendees  n=721 | |  |
| --- | --- | --- | --- | --- | --- |
|  | n | (%) | n | (%) | p |
| Gender |  |  |  |  | 0.027 |
| Women | 991 | (75) | 504 | (70) |  |
| Men | 340 | (25) | 217 | (30) |  |
| Age, years |  |  |  |  | <0.001 |
| 65-79 | 469 | (35) | 129 | (18) |  |
| 80-89 | 688 | (52) | 371 | (51) |  |
| ≥90 | 174 | (13) | 221 | (31) |  |
| Fracture type |  |  |  |  | 0.231 |
| Neck of femur | 839 | (63) | 427 | (59) |  |
| Pertrochanteric | 413 | (31) | 245 | (34) |  |
| Subtrochanteric | 79 | (6) | 49 | (7) |  |
| MNA-SF |  |  |  |  | <0.001 |
| Normal nutrition 12-14 | 612 | (46) | 167 | (23) |  |
| At risk of malnutrition 8-11 | 359 | (27) | 265 | (37) |  |
| Malnourished 0-7 | 40 | (3) | 64 | (9) |  |
| Unknown | 320 | (24) | 225 | (31) |  |
| Living arrangements* |  |  |  |  | <0.001 |
| Home | 670 | (50) | 173 | (24) |  |
| Home with home care | 385 | (29) | 203 | (28) |  |
| Assisted care facility | 124 | (9) | 156 | (22) |  |
| Institution | 146 | (11) | 184 | (26) |  |
| ASA |  |  |  |  | <0.001 |
| Grade 1-2 | 229 | (17) | 39 | (5) |  |
| Grade 3 | 860 | (65) | 411 | (57) |  |
| Grade 4-5 | 223 | (17) | 248 | (34) |  |
| Unknown | 19 | (1) | 23 | (3) |  |
| Number of medication* |  |  |  |  | <0.001 |
| <4 | 259 | (20) | 77 | (11) |  |
| 4-10 | 857 | (64) | 452 | (62) |  |
| >10 | 215 | (16) | 190 | (26) |  |
| Diagnosis of  cognitive disorder* |  |  |  |  | <0.001 |
| No | 1011 | (76) | 466 | (65) |  |
| Yes | 318 | (24) | 249 | (35) |  |
| Mobility level before hip fracture* |  |  |  |  | <0.001 |
| Unassisted outdoors | 864 | (65) | 202 | (28) |  |
| Assisted outdoors,unassisted indoors | 410 | (31) | 418 | (58) |  |
| Assisted indoors | 42 | (3) | 68 | (9) |  |
| Unable to walk | 15 | (1) | 22 | (3) |  |
| Need of mobility aid |  |  |  |  | <0.001 |
| Without mobility aid | 597 | (45) | 183 | (26) |  |
| Needs mobility aid or unable to walk | 734 | (55) | 530 | (74) |  |

Differences between fracture types were tested using Pearson Chi-Square test or Fisher-Freeman-Halton Exact test. *Unknown results of the variables less than 10 was excluded from the table. MNA-sf =Mini Nutritional Assessment short form, ASA =the American Society of Anaesthesiologists’ classification of Physical Health
